# Supplementary material for: Thermodynamic forces from protein and water govern condensate formation of an intrinsically disordered protein domain
Source: Nat Commun. 2023 Sep 21;14:5892. doi: 10.1038/s41467-023-41586-y (PMC10514047; doi:10.1038/s41467-023-41586-y)
Supplement: Supplementary file 1 — Supplementary Information [file 41467_2023_41586_MOESM1_ESM.pdf]

Thermodynamic Forces from Protein and Water Govern Condensate Formation of an Intrinsically Disordered Protein Domain

Supplementary Information

Saumyak Mukherjee and Lars V. Schäfer*

Theoretical Chemistry, Ruhr University Bochum, D-44780 Bochum, Germany

E-mail: lars.schaefer@ruhr-uni-bochum.de

Linear fit of entropy data

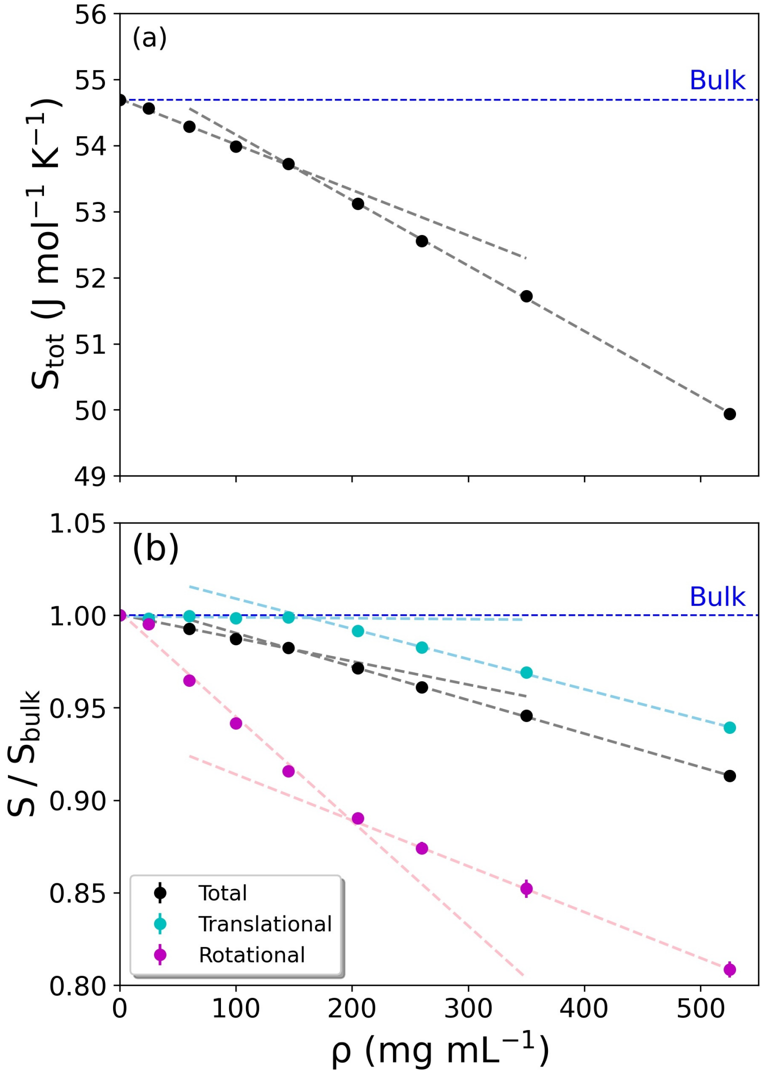

Supplementary Figure 1: Entropy of water as a function of protein concentration. Linear fits to the data are shown as dahsed lines. (a) Total entropy of water, (b) Translational, rotational and total entropy of water, each normalized with respect to bulk water.

Distributions of radii of gyration

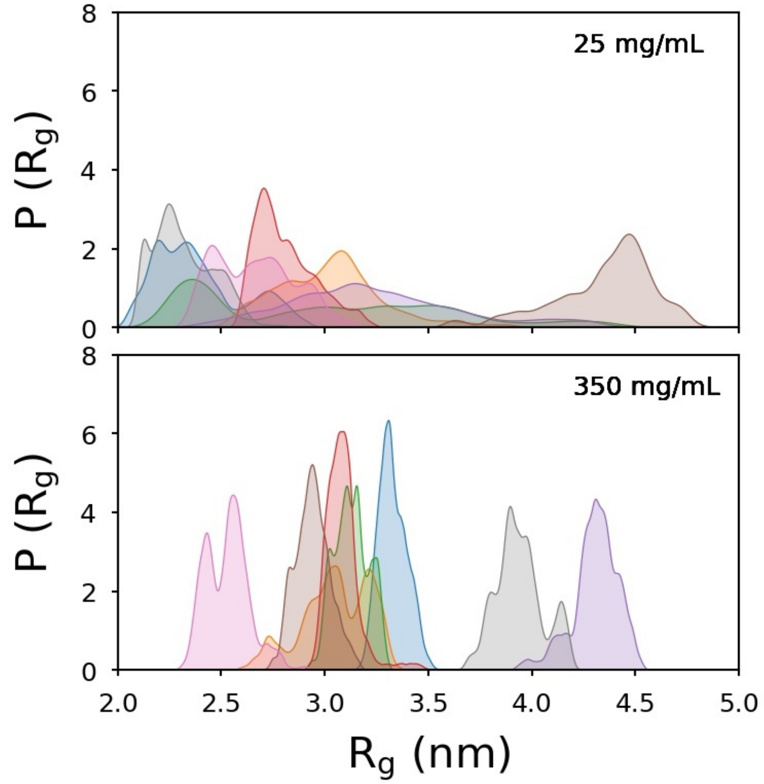

Supplementary Figure 2: Distributions of the radii of gyration of the 8 FUS-LCD proteins present in the simulation boxes of the dilute (25 mg mL^{-1}) and the dense (350 mg mL^{-1}) systems. The distributions shown are based on the three 100 ns repeats, plus the data from the two trajectories that were extended to 400 ns.

Water Entropy from Thermodynamic Integration/Free Energy Perturbation

From the total number of 177382 water molecules in the simulated cubic box of bulk (neat) a99sb-disp water, a single water molecule was randomly selected and thermodynamic integration was performed. This procedure, including the random selection of the perturbed water molecule, was repeated 100 times. To decouple the selected water molecule from the environment, initially the Coulombic interactions were turned off by changing the coupling parameter λ from 0 to 1 in discrete steps of 0.1. This was followed by changing the van der

Waals interactions in λ-steps of 0.05. A soft-core Lennard Jones potential was used with a scaling factor of 0.5. The equations of motion were integrated using Langevin dynamics with time-steps of 2 fs. The negative value of the obtained free energy of vaporization, averaged over the 100 repeats, gives the solvation free energy ΔG_{solv}.

From thermodynamic integration, we obtained the value of ΔG_{solv} = -23.45 kJ mol^{-1} for neat (bulk) a99SB-disp water at 300 K and 1 bar. The solvation free energy includes an analytical correction because of the difference in the reference states of the solvated and isolated (in vacuum) water molecules. The standard solvation free energy (and the standard enthalpy of vaporization etc.) describes the transfer of the solute (here, a water molecule) from a thermodynamic standard state of the gas (ideal gas at 1 bar) to the thermodynamic standard state in solution (ideal solution at 1 mol L^{-1}). This includes a change in concentration from 1 bar (that is, 1 mol per 24.94 L at 300 K) to 1 mol L^{-1}, which is not included in the TI calculation. The correction term is $w = -\int_{V_1}^{V_2} p\,dV = -RT\int_{V_1}^{V_2} \frac{1}{V} dV = -RT\ln(V_2/V_1) =$ 8.023 kJ mol^{-1} and the corrected solvation free energy is ΔG_{solv} = -31.47 kJ mol^{-1}. Hence, the vaporization free energy ΔG_{vap}= 31.47 kJ mol^{-1}.

The vaporization enthalpy is given by $\Delta H_{vap} = -E_{pot} + RT$, where E_{pot} is the average interaction energy of a water molecule with its surrounding in bulk water. The obtained E_{pot} from the simulation at a99SB-disp water is -52.53 kJ mol^{-1}. Since the water model used has fixed point charges, this potential energy needs to be corrected for the self-polarization, $\Delta E_{pol} = (\mu - \mu_0)^2)/2\alpha$. The magnitude of the dipole moment of the a99SB-disp water model is μ = 2.44 D and the dipole moment of a water in vacuum is μ_0 = 1.85 D. With 1 D = 3.33×10^{-30} C·m and the polarizability of water of $\alpha = 1.608 \times 10^{-40}$ F·m, one obtains ΔE_{pol} = 7.23 kJ mol^{-1}. Hence the corrected energy per water molecule is -45.30 kJ mol^{-1} and with RT = 2.48 kJ mol^{-1} one obtains ΔH_{vap} = 47.78 kJ mol^{-1}.

The entropy of water is thus obtained as $S = (\Delta H_{vap} - \Delta G_{vap})/T$. The entropy at 300 K is 54.4 J mol^{-1} K^{-1}, in excellent agreement with the 2PT entropy of 54.7 J mol^{-1} K^{-1}.

Tables for changes in water population and thermodynamic quantities

This section tabulates the data plotted in Figures 4, 7, and 8 in the main text. See also Source Data files.

Supplementary Table 1: Change in number of water molecules per protein.

ρ ($mg\ mL^{-1}$)	N_W^{rele}	N_W^{reta}
25	36039	2064
60	13623	2064
100	6845	2064
145	3905	2064
170	2934	2064
205	1941	2064
260	956	2064
350	0	2064

Supplementary Table 2: Change in total solvation entropy.

ρ ($mg\ mL^{-1}$)	$-T\Delta S_{solv}^{rele}$ ($kJ\ mol^{-1}$)	$-T\Delta S_{solv}^{reta}$ ($kJ\ mol^{-1}$)	$-T\Delta S_{solv}^{tot}$ ($kJ\ mol^{-1}$)
25	-1749.7	1738.2	-11.5
60	-1713.5	1578.9	-134.6
100	-1443.6	1403.2	-40.4
145	-1135.4	1238.5	103.1
205	-913.2	867.6	-45.6
260	-611.9	517.7	-94.3
350	0.0	0.0	0.0

Supplementary Table 3: Change in protein-water entropy.

ρ ($mg\ mL^{-1}$)	$-T\Delta S_{PW}^{rele}$ ($kJ\ mol^{-1}$)	$-T\Delta S_{PW}^{reta}$ ($kJ\ mol^{-1}$)	$-T\Delta S_{PW}^{tot}$ ($kJ\ mol^{-1}$)
25	-13789.5	12872.5	-917.1
60	-12452.5	11775.8	-676.7
100	-10919.2	10370.1	-549.1
145	-9144.8	8829.9	-314.9
205	-6817.2	6414.6	-402.6
260	-4477.5	3998.1	-479.4
350	0.0	0.0	0.0

Supplementary Table 4: Change in water-water energy.

ρ ($mg\ mL^{-1}$)	ΔE_{WW}^{rele} ($kJ\ mol^{-1}$)	ΔE_{WW}^{reta} ($kJ\ mol^{-1}$)	ΔE_{WW}^{tot} ($kJ\ mol^{-1}$)
25	-12039.8	11134.3	-905.6
60	-10739.0	10196.9	-542.1
100	-9475.6	8966.9	-508.7
145	-8009.4	7591.4	-418.0
205	-5904.0	5547.1	-356.9
260	-3865.6	3480.5	-385.1
350	0.0	0.0	0.0

Supplementary Table 5: Change in protein-water energy.

ρ ($mg\ mL^{-1}$)	ΔE_{PW}^{rele} ($kJ\ mol^{-1}$)	ΔE_{PW}^{reta} ($kJ\ mol^{-1}$)	ΔE_{PW}^{tot} ($kJ\ mol^{-1}$)
25	2791.4	-2611.9	179.5
60	2475.1	-2396.8	78.3
100	2189.7	-2111.5	78.2
145	1846.3	-1796.1	50.1
205	1371.8	-1313.4	58.4
260	903.6	-821.4	82.2
350	0.0	0.0	0.0

Supplementary Table 6: Change in total solvation enthalpy.

ρ ($mg\ mL^{-1}$)	ΔH_{solv}^{rele} ($kJ\ mol^{-1}$)	ΔH_{solv}^{reta} ($kJ\ mol^{-1}$)	ΔH_{solv}^{tot} ($kJ\ mol^{-1}$)
25	-9248.4	8522.4	-726.0
60	-8263.9	7800.2	-463.7
100	-7285.8	6855.4	-430.5
145	-6163.2	5795.3	-367.9
205	-4532.2	4233.7	-298.5
260	-2962.0	2659.0	-302.9
350	0.0	0.0	0.0

Supplementary Table 7: Change in solvation free energy.

ρ ($mg\ mL^{-1}$)	ΔG_{solv}^{rele} ($kJ\ mol^{-1}$)	ΔG_{solv}^{reta} ($kJ\ mol^{-1}$)	ΔG_{solv}^{tot} ($kJ\ mol^{-1}$)
25	-10998.1	10260.6	-737.5
60	-9977.4	9379.0	-598.4
100	-8729.4	8258.6	-470.9
145	-7298.5	7033.8	-264.8
205	-5445.4	5101.3	-344.2
260	-3573.9	3176.7	-397.2
350	0.0	0.0	0.0

Supplementary Table 8: Change in protein-protein energy.

ρ ($mg\ mL^{-1}$)	ΔE_{PP} ($kJ\ mol^{-1}$)
25	-815.5
60	-430.4
100	-358.0
145	-175.9
205	-233.0
260	-307.4
350	0.0

Supplementary Table 9: Change in protein conformational entropy

ρ ($mg\ mL^{-1}$)	$-T\Delta S_P$ ($kJ\ mol^{-1}$)
25	165.3
60	117.5
100	97.8
145	88.8
205	115.4
260	97.8
350	0.0

Supplementary Table 10: Change in total free energy

ρ ($mg\ mL^{-1}$)	ΔG ($kJ\ mol^{-1}$)
25	-1387.8
60	-911.3
100	-731.1
145	-351.9
205	-461.8
260	-606.8
350	0.0

Water population and thermodynamic changes with a FUS-LCD condensate concentration of 525 mg mL^{-1}

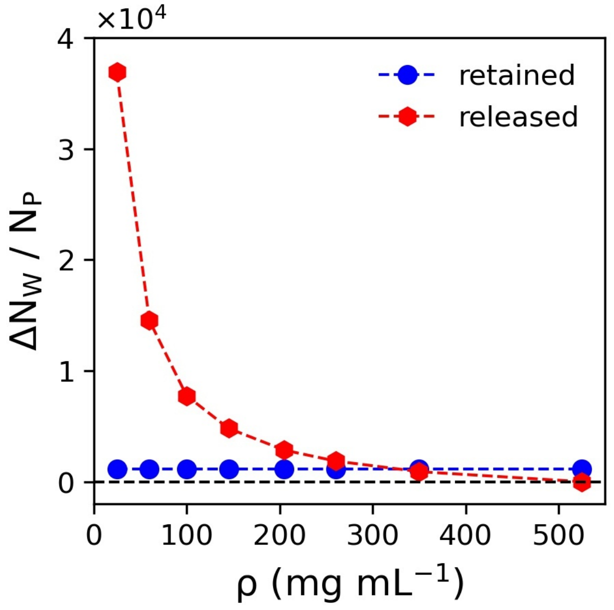

Supplementary Figure 3: Number of released and retained water molecules per protein molecule as a function of protein concentration (ρ). The number of released molecules decreases (red line) as ρ approaches the condensate concentration of 525 mg mL^{-1}, where every protein is solvated by 1159 water molecules, which are referred to as the retained waters (blue line).

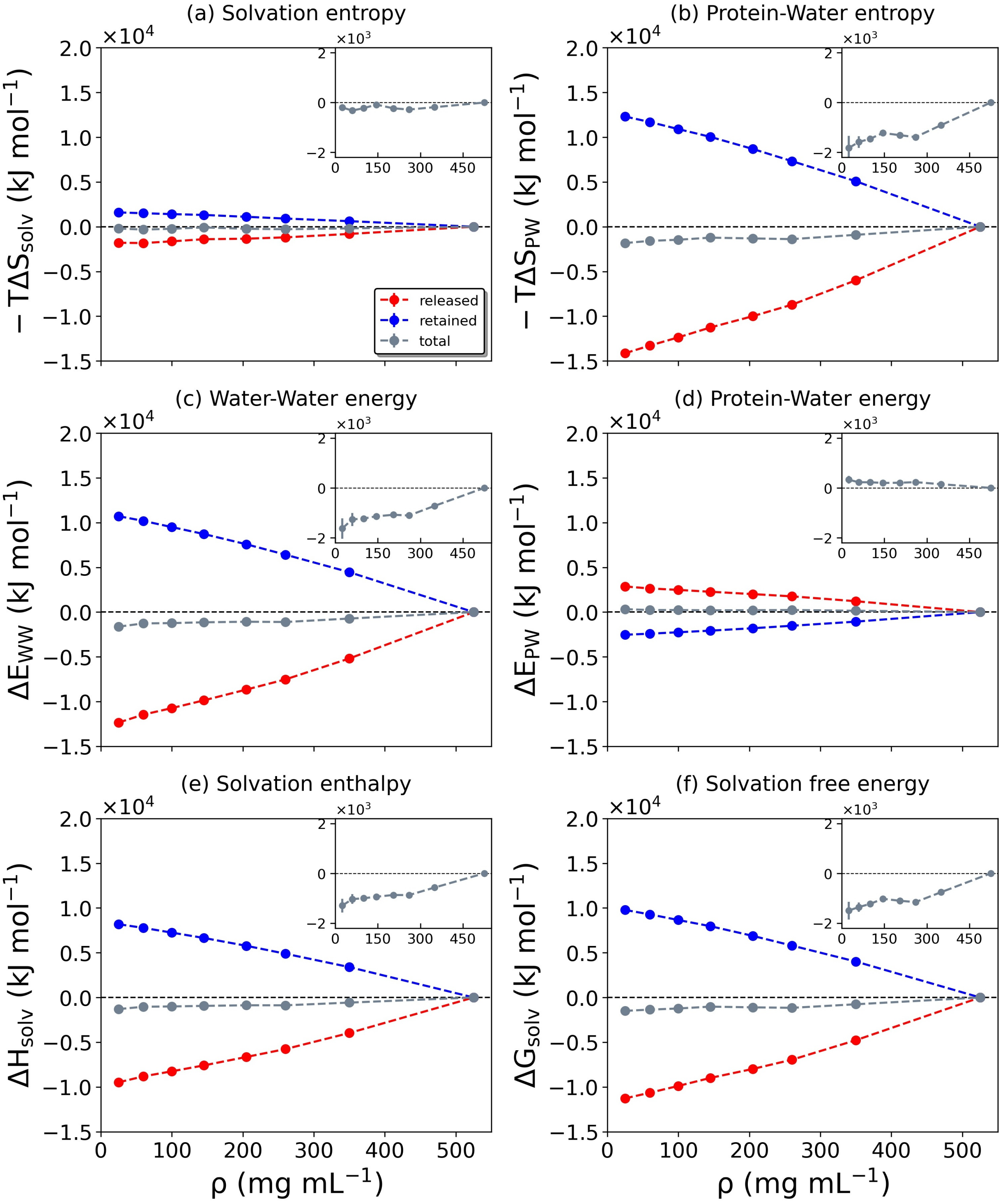

Supplementary Figure 4: Changes of the solvation-related thermodynamic quantities. The quantities plotted in (a)-(f) are indicated at the top of each panel. The dashed red, blue, grey lines denote the released, retained, and total water contributions, respectively. Data are presented as mean values ± standard deviation (SD) over three repeat simulations.

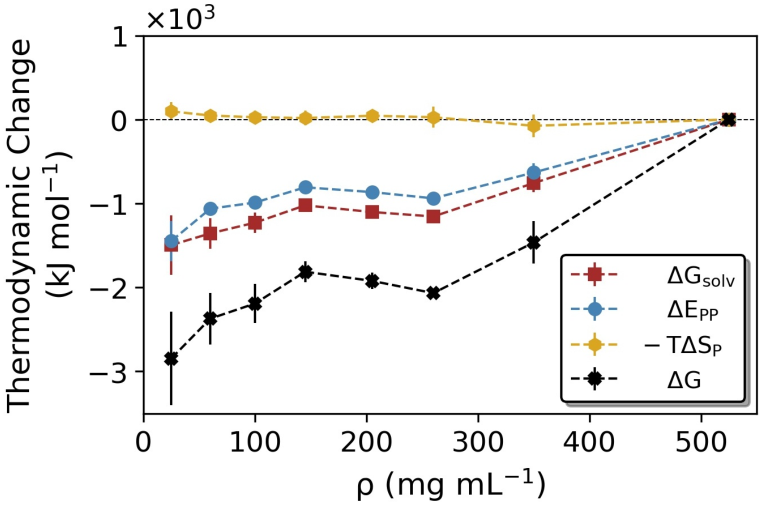

Supplementary Figure 5: Thermodynamic driving forces of FUS-LCD condensate formation. The contributions from changing total solvation free energy (ΔG_{solv}, brown dashed line) protein-protein interaction energy (ΔE_{PP}, blue), and protein conformational entropy (ΔS_P, orange) are plotted together with the resulting total free energy change ($\Delta G = \Delta G_{solv} + \Delta E_{PP} - T\Delta S_P$, black). Data are presented as mean values ± standard deviation (SD) over three repeat simulations.

Software/Codes used in this work

Supplementary Table 11: Software/Codes used in this work.

Name of the code	Version	Function	Source
DoSPT	0.2.2	Water entropy	www.dospt.org
pdb2entropy	n/a	Protein conformational entropy	https://github.com/federico-fogolari/pdb2entropy
VMD	1.9.4a9	Visualization	https://ks.uiuc.edu/Research/vmd
gmx mdrun -rerun<br>gmx energy<br>gmx hbond<br>gmx hydorder<br>gmx gyrate	2020.1	Interaction energies<br>Interaction energies<br>number of hydrogen bonds<br>tetrahedral order parameter<br>radius of gyration	https://www.gromacs.org/
