# Supplementary material for: Thermodynamic forces from protein and water govern condensate formation of an intrinsically disordered protein domain
Source: Nat Commun. 2023 Sep 21;14:5892. doi: 10.1038/s41467-023-41586-y (PMC10514047; doi:10.1038/s41467-023-41586-y)
Supplement: Supplementary file 3 — Description of Additional Supplementary Files [file 41467_2023_41586_MOESM3_ESM.pdf]

Description of Additional Supplementary Files

Supplementary Data 1: The initial and final coordinates of the MD simulation runs as well as the MD-parameter files and force field topology files to be used with GROMACS v.2020.1.
